# Diverse clinical presentation of primary spontaneous pneumothorax in patients with pectus excavatum

**DOI:** 10.3389/fsurg.2023.1245049

**Published:** 2023-08-22

**Authors:** June Lee, Jin Yong Jeong, Jong Hui Suh, Chan Beom Park, Dulee Kim, Soo Seog Park

**Affiliations:** ^1^ Department of Thoracic and Cardiovascular Surgery, Seoul St. Mary's Hospital, College of Medicine, The Catholic University of Korea, Seoul, Republic of Korea; ^2^ Department of Thoracic and Cardiovascular Surgery, Incheon St. Mary's Hospital, College of Medicine, The Catholic University of Korea, Seoul, Republic of Korea; ^3^ Department of Anesthesiology, Incheon St. Mary’s Hospital, College of Medicine, The Catholic University of Korea, Seoul, Republic of Korea

**Keywords:** primary spontaneous pneumothorax, pectus excavatum, nuss procedure, minimally invasive surgery, complication

## Abstract

**Objective:**

Patients with primary spontaneous pneumothorax (PSP) tend to be young, tall, and thin, as do those with pectus excavatum (PE). Notably, the Haller index, which measures the severity of PE, tends also to be higher in patients with PSP, further suggesting a potential predisposing factor for the development of PSP in individuals with PE. This study aimed to share clinical experiences with case series of concomitant PSP and PE and to emphasize the importance of evaluating these two conditions together.

**Methods:**

In this single-center study, we conducted a retrospective records review to identify patients who were diagnosed and treated (including surgical or conservative treatment and follow-up observation) for the diagnosis of PE between July 2011 and February 2023. From these, we selected patients who were diagnosed with both PE and PSP and analyzed their clinical presentations.

**Results:**

Among a total of 139 patients with PE, there were 8 (5.76%) who had concurrent diagnoses of PE and PSP and who underwent surgery for PSP, PE, or both. The average age of these 8 patients (male:female = 7:1) was 19.38 years. The 8 patients were grouped into four categories based on their clinical scenarios. Group A had 1 patient with PE diagnosed first, followed by the discovery of PSP during evaluation; Group B included 2 patients initially presenting with PSP and subsequently diagnosed with PE during evaluation; Group C consisted of 1 patient who had PSP before undergoing surgical PE correction; and Group D comprised 4 patients who developed PSP after PE correction.

**Conclusions:**

The incidence of PSP in patients with PE was 5.76% (8 out of 139 patients), indicating the importance of vigilant monitoring for PSP prior to PE surgery, and vice versa. Furthermore, the authors recommend close observation for PSP independent of PE surgery, even in the absence of postoperative complications.

## Introduction

Pectus excavatum (PE) is the most common congenital chest wall deformity and is characterized by a concave depression in the middle or lower sternum and in the associated costal cartilages (1). PE is known to occur in approximately 0.5% of female patients and 0.3% of males (2). Techniques for PE correction have continuously evolved since the introduction of the Nuss procedure (3), and a patient-specific approach to the minimally invasive correction of PE has proven to be effective toward accomplishing high-quality repair across the entire spectrum of PE, including in adult patients (4, 5).

However, in an analysis of chest computed tomography (CT) scans in patients with PE, Huang et al. (6) have reported the presence of blebs in 26.5% of cases, and it is known that blebs are strongly associated with primary spontaneous pneumothorax (PSP). Huang's group reported that PSP occurred in 1.91% of PE patients overall, and they found that patients with a higher Haller index (HI) also had a higher incidence of blebs (6), suggesting that patients undergoing surgical PE correction should be checked for blebs before PE surgery. HI is a measure used to assess the severity of PE in patients, which is calculated by dividing the transverse diameter of the chest by the anterior-posterior distance on chest CT (7).

PSP typically occurs in young, tall, slim males, and subpleural blebs and bullae are strongly implicated the development of PSP. However, the exact cause of these blebs and bullae remains unclear (8). The reported incidence of PSP is 7.4 cases per 100,000 person-years in male patients and 1.2 cases per 100,000 person-years in females. However, the actual prevalence of PSP may be underestimated, as the number of completely asymptomatic cases is not well known (9).

Therefore, the primary purpose of this study was to share the clinical experience of cases where both PE and PSP have occurred.

## Methods

Among individuals who sought outpatient consultations at the thoracic surgery department at Incheon St. Mary's Hospital between July 2011 and February 2023, we identified 139 patients with a diagnosis of PE who underwent either surgical correction or outpatient follow-up for PE. Upon retrospective review of these cases, we discovered that 8 of these patients had concomitant PSP. Our objective was to analyze the clinical features of these 8 individuals who were diagnosed with both PE and PSP.

The patients were divided into four groups based on the order of diagnosis or treatment of PE and PSP, and we described their age (based on the timing of surgery for pneumothorax, or the time of diagnosis if surgery was not performed), gender, HI, temporal difference between the diagnosis of PE and PSP, radiological features, surgical findings, and postoperative courses of the patients.

## Results

**Figure 1:**
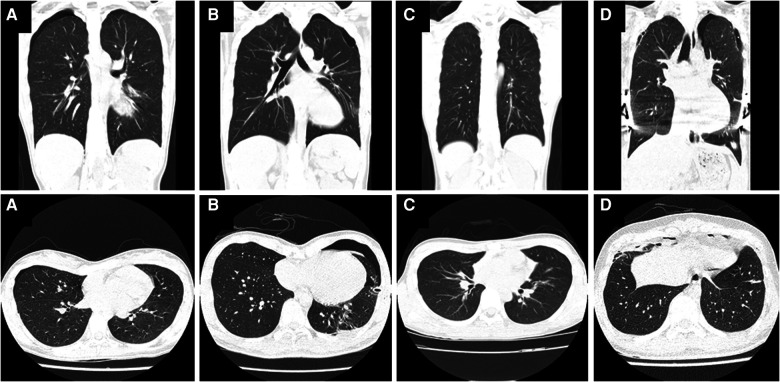
Chest computed tomography (CT) images showing primary spontaneous pneumothorax (PSP) in patients with pectus excavatum (PE). (**A,a**) Patient 1: An 18-year-old female with pneumothorax found incidentally during PE evaluation. (**B,b**) Patient 2: A 21-year-old male with PE found during evaluation and treatment of PSP. (**C,c**) Patient 4: A 15-year-old male with confirmed PE whose PSP occurred before the Nuss procedure. (**D,d**) Patient 5: An 18-year-old male with bilateral PSP unrelated to the surgery after a Nuss procedure to correct PE.

A total of 8 patients (5.76%) were analyzed in this study, including 7 males and 1 female. Their characteristics and clinical course details are summarized in Table 1. The average age of the patients was 19.38 years, and the mean HI value was 4.12. The 8 patients were grouped into four categories based on their clinical scenarios. Group A consisted of 1 patient who was initially diagnosed with PE, followed by the discovery of PSP during evaluation of PE. In Group B, 2 patients were initially diagnosed with PSP and were found to have PE almost immediately during evaluation of PSP. Group C included 1 patient with known PE who experienced PSP prior to undergoing surgical PE correction. Lastly, Group D comprised 4 patients who developed PSP after (and not as a direct complication of) PE correction. Representative CT images of PSP accompanying PE in each group are shown Figure 1.

**Table 1:** The demographic data of all 8 patients.

Group	Patients	Sex	Age (years)	Ht (cm)	Wt (kg)	BMI (kg/m²)	HI	Primary diagnosis	Time interval between the diagnoses of the two conditions	Repair of PE	Surgery for PSP
A	1	F	18	170.0	46.2	15.99	4.80	PE	After a consultation for PE, the patient was immediately diagnosed with PSP upon imaging on the same day.	−	+
B	2	M	21	180.5	69.3	21.27	4.76	PSP	During the process of diagnosing PSP, PE was immediately detected on the same day through imaging examination.	+	+
	3	M	17	181.1	69.9	21.31	3.31	PSP		+	+
C	4	M	15	176.7	64.5	20.66	3.96	PE	PSP occurred 3 years after being diagnosed with PE (6 days before the Nuss procedure).	+	+
D	5	M	18	178.2	50.1	15.78	4.28	PE	PSP occurred the day after repair of PE.	+	−
	6	M	33	178.4	70.2	22.06	5.58	PE	PSP occurred 6 months after repair of PE.	+	−
	7	M	20	183.7	69.2	20.51	3.63	PE	PSP occurred 17 months after repair of PE.	+	+
	8	M	13	167.6	42.6	15.17	2.67	PE & PC	PSP occurred 4 months after repair of PE & PC.	+	+

Ht, height; Wt, weight; BMI, body mass index; HI, Haller index; PE, pectus excavatum; PSP, primary spontaneous pneumothorax; PC, pectus carinatum.

### Group A

Patient 1 was an 18-year-old female patient with funnel chest. When she presented for evaluation of PE, the chest x-ray and CT revealed a right-sided pneumothorax. There was a bleb at the right lung apex, but because it was her first episode of PSP, a watchful waiting approach was taken. However, the right-sided PSP recurred about a month later, and video-assisted thoracoscopic surgery (VATS) was performed. It was decided in consultation with the patient not to proceed with surgery for PE correction. Following the VATS for pneumothorax, the patient was discharged 8 days after the surgery.

### Group B

Patient 2, a 21-year-old male, presented with left-sided chest pain and was diagnosed with pneumothorax. PE was diagnosed on CT imaging following the insertion of a left chest tube. CT showed bilateral blebs, but the pneumothorax was only present on the left side. Because of persistent air leakage, the patient underwent left-sided surgery for the pneumothorax and was discharged after 6 days. Two months later, he returned for surgical correction of the PE.

Patient 3, a 17-year-old male, presented with right-sided chest pain and was diagnosed with both pneumothorax and PE during the initial evaluation. Chest CT revealed blebs on both apices. During the follow-up period, the patient had a recurrent right-sided PSP, which was treated surgically, resulting in discharge after 4 days. One year later, the patient received simultaneous surgical treatment for PE and left-sided pneumothorax.

### Group C

Patient 4, a 15-year-old male, had been under observation for 3 years after a diagnosis of PE and was scheduled for a Nuss procedure. However, 6 days before the surgery, he suddenly developed left-sided PSP. As a result, he underwent surgery for both PE and PSP and was discharged on postoperative day 10.

### Group D

Patient 5, an 18-year-old male, underwent Nuss procedure for PE using a 2 mm thoracoscope, and it was confirmed with the thoracoscope that there were no organ injuries, including the lungs, after the insertion and fixation of the pectus bar. He experienced bilateral PSP the day after the procedure. Blebs had been observed in both apices prior to the surgery. The PSP was treated by chest tube insertion for 12 days. The patient was discharged and there was no recurrent pneumothorax during more than 2 years of follow-up.

Patient 6, a 33-year-old male, was discharged without any significant complications after the repair of PE, but returned 6 months later with respiratory distress and was diagnosed with bilateral PSP. CT scans revealed no prominent blebs in the patient from the beginning, so he was treated with only chest tube insertion for 5 days before discharge.

Patient 7, a 20-year-old male, who underwent the Nuss procedure, was diagnosed with bilateral PSP during outpatient follow-up after 17 months. As there was no improvement, the patient underwent bilateral VATS wedge resection and was discharged 6 days later.

Patient 8, a 13-year-old male, presented to the emergency department with respiratory distress and chest pain 4 months after diagnosis and surgical correction of PE and rebound pectus carinatum. He was diagnosed with bilateral tension pneumothorax in a buffalo chest. Thoracoscopy revealed multiple bullae in the upper lobe of the right lung, which we identified as the cause of the tension pneumothorax. Right pulmonary wedge resection was performed, and the patient was discharged without complication after 3 days.

## Discussion

We have found several studies on the radiological correlation between PSP and PE. However, there is a lack of specific research describing the actual clinical course of patients with simultaneous PE and PSP. Patients with PE were reported to have a significantly higher cumulative incidence of PSP that was approximately twice that (0.36% vs. 0.15%; hazard ratio 7.83 and *p* = 0.002) of a control group (10). Additionally, Kılıçgün et al. (11) found that patients with PSP had a significantly higher mean HI (2.41 vs. 2.09, *p* = 0.006) compared to those in the control group. Moreover, Peters et al. (12) reported that PSP was associated with a longer and flatter chest shape, and Park et al. (13) showed that PSP patients had anteroposteriorly flatter, laterally narrower, and craniocaudally taller thoraxes.

Patients with PE need to be aware that PSP unrelated to surgical complication may occur after surgical correction of PE. In the general population, the incidence of PSP is reported, 1.2–7.4 cases per 100,000 person-years (9). Huang et al. (6) have reported that among patients with PE, PSP occurred in 5.6% of those with blebs and in 0.5% of those without blebs. This supports the idea that pneumothorax can occur in patients with PE in the presence or absence of blebs, suggesting that individuals with PE carry a potential risk of pneumothorax.

When assessing the risk of PSP in patients with PE based on the presence or absence of blebs, it is important to know that the sensitivity of CT scans for detecting blebs may not be very high, and varies widely, ranging from 57.6% to 97.0%, depending on the type of CT (14, 15). In other words, since CT cannot diagnose blebs with 100% certainty, it is important to consider the possibility of PSP when contemplating surgical treatment for patients with PE.

The surgical outcomes of PE correction have been progressively improving because of advances in minimally invasive surgical techniques (5). However, the chest wall corrected by the Nuss procedure becomes more vulnerable to pneumothorax, and there are several reports of life-threatening cases of bilateral PSP in a buffalo chest following the Nuss procedure for PE (16–18). The potential risk involves the development of bilateral pneumothorax in a buffalo chest due to bilateral thoracic cavity communication following the Nuss bar insertion, which can have catastrophic consequences if not promptly and appropriately managed surgically. The case of Patient 8 can be considered the most cautionary in this regard. This patient has previously been reported as a case study (16) in our institution. He presented with PSP unrelated to the insertion of the Nuss bar, which progressed to a life-threatening bilateral tension pneumothorax. After experiencing this case, we recognized the need for the present study on PE and PSP and were motivated to plan it.

VATS is a well-established, highly effective approach for surgical treatment of PSP (19). Considering the usefulness of thoracoscopy, although it may not be applicable to all patients, utilizing thoracoscopy when performing surgical PE correction can facilitate the identification and resection of blebs. Particularly, the use of thoracoscopy during PE correction can also help identify rare complications, such as lung entrapment by the Nuss bar, making it a beneficial approach without major drawbacks. We have also used a 2 mm thoracoscope during the Nuss procedure to minimize the occurrence of organ injuries, including the heart and lungs.

Taking all of this into consideration, our institution has recently been using pre- and post-operative CT scans during PE correction to check for the presence of blebs. We also alert patients and caregivers to the risk PSP in PE. We anticipate that the assessment for the presence of blebs by thoracoscopy or similar methods in patients undergoing the Nuss procedure for PE may further improve the prediction of PSP in patients with PE.

Limitations of this study include its retrospective nature involving a medical chart review and the small sample size. Additionally, our findings are based solely on shared clinical experiences, and we did not obtain statistically significant results. Further research is warranted to explore the clinical relationship between PSP and PE in greater depth.

## Conclusion

In conclusion, we have compiled the clinical features of patients who were diagnosed with both PSP and PE, taking into account the well-known association between the two conditions. When managing patients with PE, it is crucial to consistently keep in mind the potential presence of PSP and to be ready to manage both conditions simultaneously when required.

## Data Availability

The original contributions presented in the study are included in the article/Supplementary Material, further inquiries can be directed to the corresponding author.
